# Study on the nitrogen content estimation model of cotton leaves based on “image-spectrum-fluorescence” data fusion

**DOI:** 10.3389/fpls.2023.1117277

**Published:** 2023-03-01

**Authors:** Shizhe Qin, Yiren Ding, Zexuan Zhou, Meng Zhou, Hongyu Wang, Feng Xu, Qiushuang Yao, Xin Lv, Ze Zhang, Lifu Zhang

**Affiliations:** ^1^Xinjiang Production and Construction Crops Oasis Eco-Agriculture Key Laboratory, Shihezi University College of Agriculture, Shihezi, China; ^2^Aerospace Information Research Institute, Chinese Academy of Sciences, Beijing, China

**Keywords:** cotton, nitrogen, hyperspectral, chlorophyll fluorescence, digital images, data fusion

## Abstract

**Objective:**

Precise monitoring of cotton leaves’ nitrogen content is important for increasing yield and reducing fertilizer application. Spectra and images are used to monitor crop nitrogen information. However, the information expressed using nitrogen monitoring based on a single data source is limited and cannot consider the expression of various phenotypic and physiological parameters simultaneously, which can affect the accuracy of inversion. Introducing a multi-source data-fusion mechanism can improve the accuracy and stability of cotton nitrogen content monitoring from the perspective of information complementarity.

**Methods:**

Five nitrogen treatments were applied to the test crop, Xinluzao No. 53 cotton, grown indoors. Cotton leaf hyperspectral, chlorophyll fluorescence, and digital image data were collected and screened. A multilevel data-fusion model combining multiple machine learning and stacking integration learning was built from three dimensions: feature-level fusion, decision-level fusion, and hybrid fusion.

**Results:**

The determination coefficients (R^2^) of the feature-level fusion, decision-level fusion, and hybrid-fusion models were 0.752, 0.771, and 0.848, and the root-mean-square errors (RMSE) were 3.806, 3.558, and 2.898, respectively. Compared with the nitrogen estimation models of the three single data sources, R^2^ increased by 5.0%, 6.8%, and 14.6%, and the RMSE decreased by 3.2%, 9.5%, and 26.3%, respectively.

**Conclusion:**

The multilevel fusion model can improve accuracy to varying degrees, and the accuracy and stability were highest with the hybrid-fusion model; these results provide theoretical and technical support for optimizing an accurate method of monitoring cotton leaf nitrogen content.

## 1 Introduction

Cotton is the dominant industry in the Xinjiang region, China’s largest production base of high-quality commodity cotton. In 2022, the cotton planting area covered approximately 83% of the farmland in Xinjiang, with over 90% production. Nitrogen is an indispensable life element of cotton crops. It is an important component of protein, nucleic acid, chlorophyll, and some hormones, provides important support for photosynthesis, and significantly impacts crop growth and development (Han et al., 2022; Wang et al., 2022). Nitrogen fertilization is the most used fertilizer type during cotton cultivation and management, with the highest application rate (70–80%) used in drip irrigation. Proper nitrogen fertilizer application can promote cotton growth and increase yield, but unreasonable application reduces fertilizer efficiency and causes fertilizer waste and serious environmental pollution problems (Zhang et al., 2018). Therefore, it is important to understand the nitrogen nutrition of cotton in real-time during the growth process and to develop an efficient and scientific nitrogen monitoring method to improve nitrogen fertilizer efficiency.

Traditional nitrogen monitoring methods are expensive, destructive, and time consuming. With the development of modern information technology, efficient and non-destructive monitoring methods, such as hyperspectral and digital imaging, have become increasingly popular worldwide. For example, researchers screened sensitive bands based on hyperspectral data or combined them with vegetation indices to build and apply nitrogen nutrition inversion models for different crops (Yao et al., 2010; Jin et al., 2013; Zhao et al., 2018; Luo et al., 2020; Zhang et al., 2021); this indicates that crop nitrogen content estimation using hyperspectral techniques is practical and feasible. Chlorophyll fluorescence monitoring differs from hyperspectral monitoring because it provides information directly related to plant physiological and functional properties (Ptushenko et al., 2014). Fluorescence signals can predict the growth status of plants and explain the changes in plant physiological and biochemical processes (Zivcak et al., 2015; Jia, 2016). Nitrogen application rate can notably affect the characteristic changes of chlorophyll fluorescence parameters when used to monitor crop nitrogen content (He et al., 2020), which indicates that fluorescence parameters are ideal indicators of crops’ nutritional status and can directly monitor nitrogen content (Feng et al., 2015; Li et al., 2020a). Compared with hyperspectral and chlorophyll fluorescence, digital image technology is simple to operate, visualize, and apply. It includes an interval of interest segmenting, converting image color space (Huang et al., 2020), extracting relevant color and texture features, and building predictive models (Xiao, 2022). In recent years, scholars have achieved good results using this technology to model and invert the nitrogen nutrients of crops such as rice (Saberioon et al., 2013; Wang et al., 2014; Li et al., 2015), corn (Li et al., 2010; Liu and Li, 2010), sugar beet (Moghaddam et al., 2010), and cotton (Zhang et al., 2020; Hong et al., 2022).

The spectra of reflection or absorption can reflect differences in crop composition and structure (Tong et al., 2006). Nitrogen limitation and nitrogen redundancy will cause, among other symptoms, yellowing or greenness of crop leaves and changes in leaf thickness and water content, resulting in changes in spectral reflectance characteristics. However, it is impossible to obtain the true color based on different color spaces, such as RGB, and shape-related values, such as area features. Nevertheless, the surface color, shape, and other feature changes of crops can be determined using digital images to reflect the nitrogen nutrition status. The changes in chlorophyll fluorescence parameters can directly or indirectly reflect the primary reaction, electron transfer, and CO_2_ assimilation process of photosynthesis and affect the energy distribution of crops through nitrogen. This causes dynamic changes in fluorescence, photosynthesis, and heat dissipation that can be used to monitor nitrogen content (Kumar et al., 2014). The three data sources can be used separately for crop nitrogen nutrient inversion. However, the nitrogen information monitoring of single data sources cannot consider the expression of the above phenotype and physiological parameters, and, currently, the monitoring accuracy cannot meet the requirements of precision agriculture. One of the keyways to solve this problem is to effectively integrate the information obtained from “spectrum, fluorescence, and image.”

In this study, nitrogen content estimation models that combine multiple machine learning and stacking integrated learning were built from the three levels of feature-level fusion, decision-level fusion, and hybrid fusion using cotton as the research object. The fusion models were compared with the three single data source models. The aim of this study was to improve the accuracy and stability of nitrogen estimation models and provide theoretical and methodological support for accurately monitoring cotton nitrogen content and efficient use of fertilization.

## 2 Materials and methods

### 2.1 Experimental design

#### 2.1.1 Experimental environment

**Figure 1:**
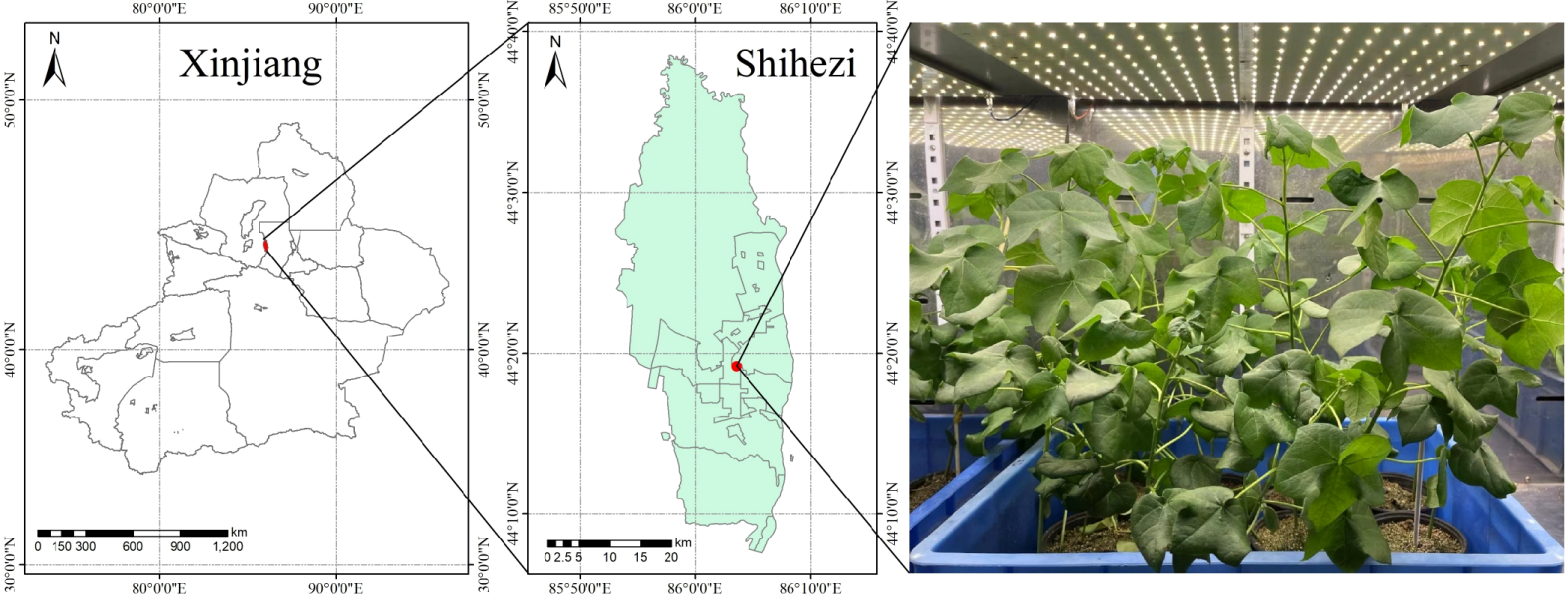
Location and environmental overview of the test area.

This experiment was performed at the College of Agronomy, Shihezi University, Shihezi City, Xinjiang Uygur Autonomous Region (E 86.06°, N 44.32°) (**Figure 1**). A laboratory incubator was set to a diurnal temperature of 30°C/26°C on a light/dark cycle of 12 h each, with a light intensity of 20,000 Lux. The potting soil was taken from the surface (0–40 cm) of the agricultural experimental field of Shihezi University (E 86.03°, N44.18°); the soil texture was medium loam, with alkaline hydrolyzable nitrogen, organic matter, available phosphorus, and available potassium contents of 103.8 mg/kg, 18.1 mg/kg, 8.91 mg/kg, and 290.3 mg/kg, respectively, and a field water holding capacity of 24.6%. After air drying and sieving, 3 kg of soil was weighed into flowerpots with a uniform upper diameter of 40 cm and a height of 30 cm.

#### 2.1.2 Experimental treatment

The cotton variety used was Xinluzao No. 53, the main local cultivar. Five nitrogen treatments (0 g/pot (N0), 1.876 g/pot (N1), 5.628 g/pot (N2), 7.504 g/pot (N3), and 9.380 g/pot (N4) of pure nitrogen) were established. Each treatment had nine repetitions, and N2 was the standard nitrogen application treatment. A total of 45 pots were planted, with two cotton plants per pot. Pure phosphorus and pure potassium were applied at 1.876 g/pot and 1.876 g/pot, respectively. The experimental fertilizers used were urea (N 46%), calcium superphosphate (P_2_O_5_ 12%), and potassium chloride (K_2_O 60%). All phosphate and potassium fertilizers were applied before sowing; 25% of the nitrogen fertilizers were applied basally, and the remaining 75% were drip-applied with water. Three drip applications were made during the cotton cultivation period. The other management schemes of the pot experiments followed local cotton cultivation management procedures.

### 2.2 Data acquisition and processing

#### 2.2.1 Data acquisition

##### 2.2.1.1 Hyperspectral data acquisition

**Figure 2:**
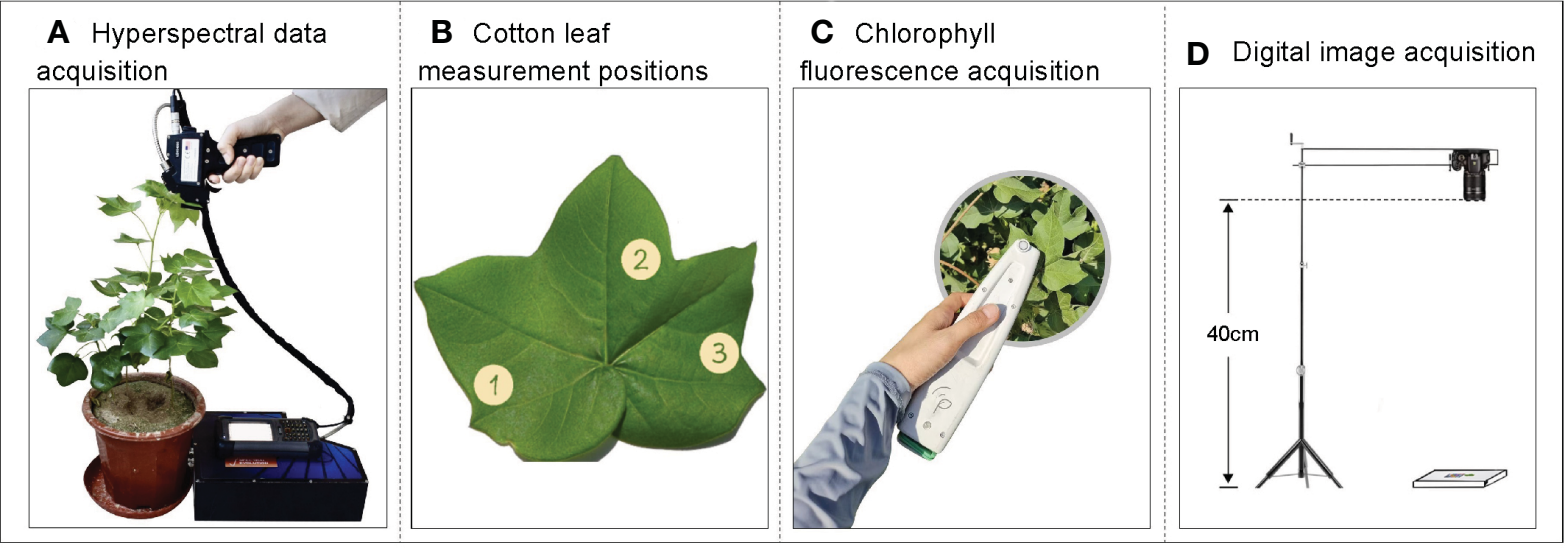
Schematic diagram of data acquisition. **(A)** Hyperspectral data acquisition, **(B)** Leaf measurement positions, **(C)** Chlorophyll fluorescence, and **(D)** digital image acquisition.

In this experiment, a portable spectral surface spectrometer SR-3500 (Spectral Evolution) was used as the hyperspectral data acquisition instrument, which has a wavelength range of 350–2500 nm at 1 nm intervals. Spectral data were collected at 10:00–14:00 local time on sunny days 60, 80, and 100 days after cotton emergence. Three main cotton stem leaves with uniform growth were selected for each treatment, and non-destructive data acquisition was conducted using the spectrometer’s leaf clamp and built-in light source (**Figure 2A**). The leaves were labeled with serial numbers. Three positions were measured for each leaf (**Figure 2B**), and the average data of each of the three measurement points were used as the raw spectral data (R) for that leaf, with whiteboard correction before measuring different leaves; care was taken to avoid leaf veins.

##### 2.2.1.2 Chlorophyll fluorescence data acquisition

The non-destructive sampling of cotton leaf fluorescence parameters was performed using a MultispeQ multifunctional phytometer (**Figure 2C**), and the measurement times were 10:00–14:00 under light adaptation and 22:00–03:00 under dark adaptation on sunny days. Three positions were measured for each leaf (**Figure 2B**) and the average was taken as the raw fluorescence data for that leaf.

##### 2.2.1.3 Image data acquisition

A Nikon D5300 digital camera was used to capture cotton leaf images from 10:00–14:00 on sunny days. Cotton leaf images were captured simultaneously as hyperspectral and fluorescence data under light adaptation to avoid errors caused by different acquisition times of data. A white background plate was used as the bottom surface to take photos, and the collection sequence of the blades was consistent with the hyperspectral and fluorescence data, which was convenient for subsequent collation and analysis. A 24-color standard color-correction card was placed on the background board for image color correction. When taking photos, the lens and leaf were vertical and kept at a fixed height of 40 cm (**Figure 2D**). The images were saved in JPEG format.

##### 2.2.1.4 Cotton leaf nitrogen data acquisition

After the spectral, fluorescence, and image data were collected, cotton leaves were harvested, stored in envelopes with ice bags, and brought to the laboratory. The cotton leaves were fixed at 105°C for 30 min, dried at 80°C to a constant weight, crushed, and then heated for digestion using the H_2_SO_4_-H_2_O_2_ method. The total nitrogen content was determined using the Kjeldahl method.

#### 2.2.2 Data processing

##### 2.2.2.1 Hyperspectral data processing method

Data processing was implemented using Python 3.8 programming, and the raw hyperspectral data were first convolutionally smoothed (Savitzky–Golay) to improve the signal-to-noise ratio. Then, the second-order differentiation of the spectral logarithm ([lg(SG)]”) was used to eliminate baseline drift and background signals to improve the analytical accuracy (Wang, 2013). Finally, a standard normal variate (SNV) and detrending were used to eliminate spectral errors caused by solid particle size, surface scattering, and light range variations (Jin et al., 2013).

Because there is homogeneity and redundancy in full-band spectral data, two preprocessing methods, SG-SNV-Detrending and [lg(SG)]”, were selected to improve the signal-to-noise ratio and analysis accuracy in this study. Pearson’s correlation analysis was conducted on the spectra obtained using different pretreatment methods and cotton leaf nitrogen content. The feature bands that significantly correlated at the 0.01 level were selected, and the redundant information was eliminated using the random-frog method to reduce the number of nitrogen-sensitive feature bands while ensuring minimum collinearity among the selected bands.

##### 2.2.2.2 Chlorophyll fluorescence data processing method

The variable fluorescence (Fv), PSII maximum photochemical efficiency (Fv’/Fm’), non-photochemical quenching coefficient (NPQ), and photochemical quenching coefficient (qL) were calculated from the parameters of initial fluorescence (F0), maximum fluorescence (Fm), steady-state fluorescence intensity (Fs), initial fluorescence under light adaptation (F0’), maximum fluorescence under light adaptation (Fm’), and variable fluorescence under light adaptation (Fv’). Chlorophyll fluorescence eigenvectors were screened using Pearson correlation analysis for subsequent modeling. The formula for calculating the fluorescence parameters is as follows:


Fv=Fm−F0

$$Fv'/Fm'=\frac{\left(Fm^{\prime }-F0'\right)}{Fm'}$$

$$NPQ=\frac{Fm}{Fm'}-1$$

$$qL=\frac{\left(Fm^{\prime }-Fs\right)}{\left(Fm'-F0'\right)}\times \frac{F0'}{Fs}$$

##### 2.2.2.3 Image data processing method

**Figure 3:**
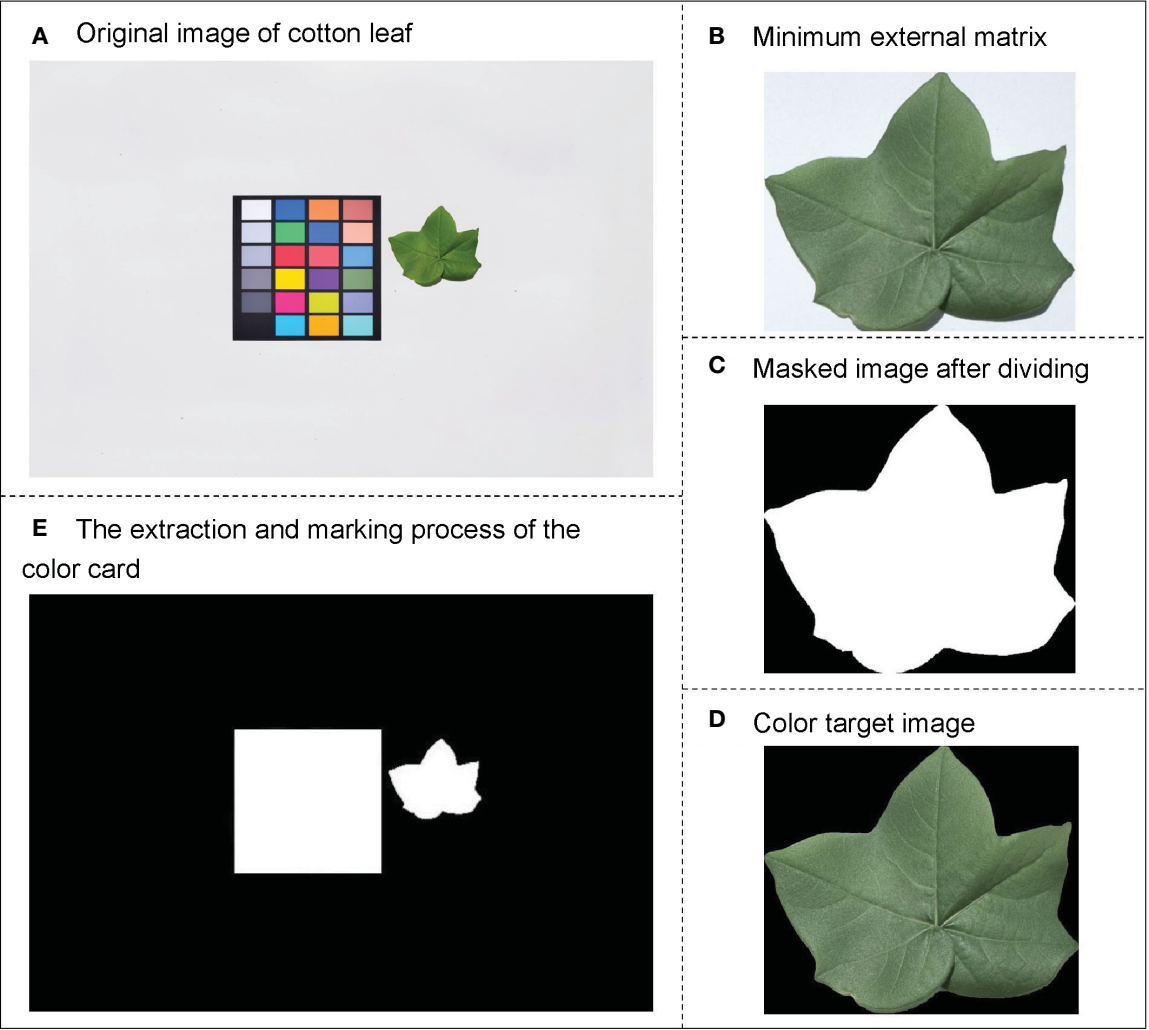
Schematic diagram of image data processing. **(A)** Original image of cotton leaf, **(B)** Minimum external matrix, **(C)** Masked image after dividing, **(D)** Color target image, and **(E)** Extraction and marking process of color card.

Cotton leaf image processing includes color correction, segmentation, and extraction of the color and morphological features of the region of interest. First, the original image (**Figure 3A**) was corrected using the 24-color-correction card, which reduces the errors caused by solar illumination or the external environment on the color features of the leaf. Then, to eliminate the influence of the color-correction card on cotton leaf segmentation, the maximum outline of the cotton leaf was found to obtain its minimum external rectangle (**Figure 3B**), the RGB (a color space, composed of red, green, and blue color channels) image was converted into HSV (a color space, composed of hue, saturation, and value channels) format, and the interval of interest to be segmented was detected using the minimum and maximum HSV values. The noise was removed using a closing operation, and the mask image was finally obtained (**Figure 3C**).

The color features of the cotton leaf images were extracted using Python-OpenCV2. First, the color target image (**Figure 3D**) was converted into different color spaces to obtain the RGB, HSV, and L*a*b* (a color space composed of a luminance channel and a and b color channels), which are used for color feature extraction of cotton leaves. Finally, nine color feature parameters, including the first-order color moment in the RGB, HSV, and L*a*b* color space of cotton leaves, were obtained.

The size of the leaf area directly determines the intensity of photosynthesis and the final yield of crops (Begonia and Begonia, 2007). Relevant studies have confirmed a significant correlation between crop leaf area and nitrogen content (Zhang, 2019). The morphological features of the cotton leaf area in this study were extracted using the grid method. First, the 24-color-correction card grid image was segmented based on the color feature information (**Figure 3E**). Because the color-correction card is a customized standard grid of 10 cm × 10 cm, the pseudo-leaf area to be measured can be represented by the number of pixels. The number of pixel points measured on the leaf (pseudo-leaf area) is multiplied by the scaling ratio (K) to calculate the actual area of the leaf, which is used as the morphological feature parameter of the leaf for subsequent modeling. K can be expressed as follows:


$$K=\frac{L}{X}$$

where *L* is the actual area of the color-correction card, which is 100 cm^2^, and *X* is the number of pixels in the pixel coordinate system of the card.

##### 2.2.2.4 Nitrogen data processing method

The total nitrogen content of all cotton leaves obtained in 2.2.1 was summarized for processing, and the data marked as NULL due to experimental mis-operation were removed as outliers. Considering the clear difference in data values under different nitrogen application levels, outliers cannot be eliminated using discretization. Therefore, the data under different nitrogen application levels were separated, and the outliers were eliminated again using the criteria of the quartile method: when the nitrogen value was less than the 25th percentile minus 1.5 times the interquartile range (IQR) or greater than the 75th percentile plus 1.5 times the IQR, the data point was eliminated as an outlier.

### 2.3 Model construction and evaluation

Of the 289 groups of cotton leaf data samples, 40, 58, 63, 69, and 59 belonged to the N0, N1, N2, N3, and N4 groups, respectively. The above data samples were randomly divided into training and validation sets using a 7:3 ratio. To evaluate the model’s generalization performance, reduce over-fitting, and obtain as much effective information as possible, all machine learning or integrated learning models adopted 5-fold cross-validation.

#### 2.3.1 Data-fusion methods

##### 2.3.1.1 Multi-source feature-level fusion method

**Figure 4:**
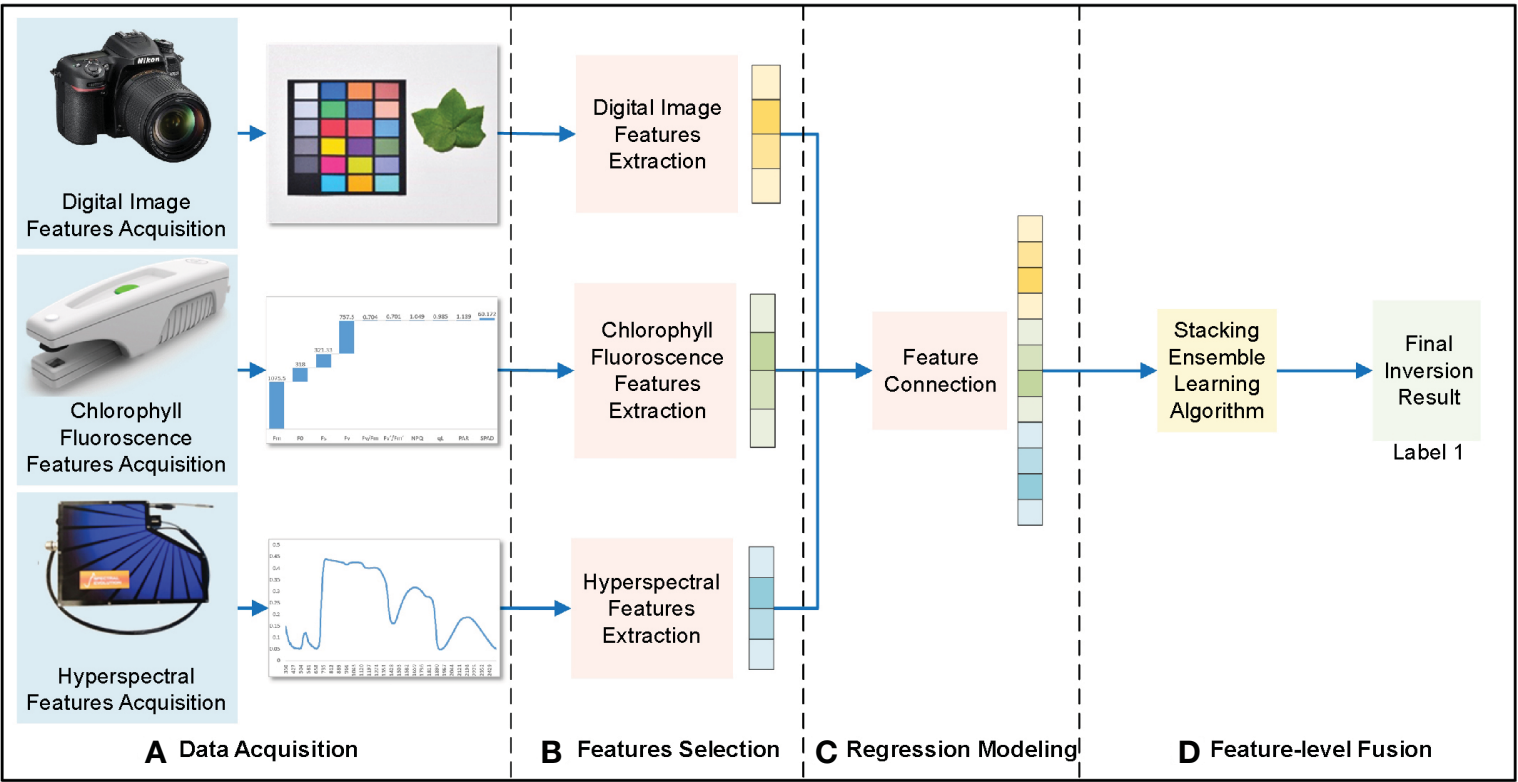
Feature-level fusion framework of cotton leaf nitrogen inversion. **(A)** Data acquisition, **(B)** Features selection, **(C)** Regression modeling, **(D)** Feature-level fusion.

Feature-level fusion extracts the features from different data sources, such as digital images, chlorophyll fluorescence, and hyperspectral data. The feature vectors extracted from these data sources were connected and substituted into the regression model to obtain inversion results (**Figure 4**).

The extracted digital image features, chlorophyll fluorescence features, and hyperspectral features were fused using the cascade method. Because integrated learning has strong noise resistance and generalization ability, the stacking integrated learning model was selected for regression prediction. This is a layered integration model; the data set was trained and predicted with the base-learner, and the output value was used as the input value for the next stage of training for the meta-learner to obtain the final forecast result. In this study, the stacking integrated learning algorithm included four learners: random forest (RF), support vector machine (SVM), K-nearest neighbor (KNN), and ridge regression (RR). Subsequently, the appropriate base- and meta-learners were selected to optimize the model fit and generalization ability to obtain the cotton leaf nitrogen inversion results through feature-level fusion.

##### 2.3.1.2 Multi-source decision-level fusion method

**Figure 5:**
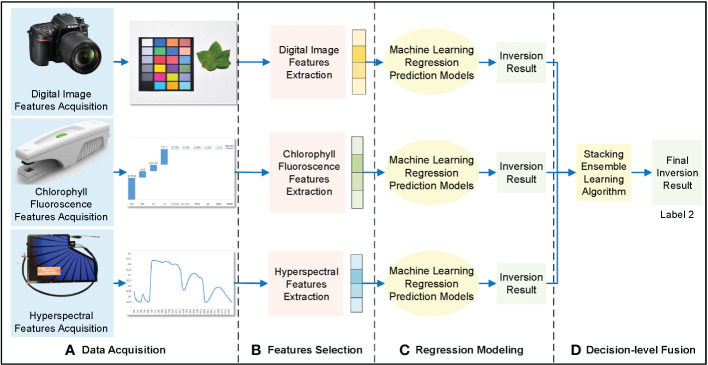
Decision-level fusion framework of cotton leaf nitrogen inversion. **(A)** Data acquisition, **(B)** Features selection, **(C)** Regression modeling, **(D)** Feature-level fusion.

Decision-level fusion separately models digital image features, chlorophyll fluorescence features, hyperspectral features, and cotton leaf nitrogen content in three dimensions based on the prediction results to match image and text data (**Figure 5**).

The features extracted from digital images, chlorophyll fluorescence, and hyperspectral data were used for initial machine learning modeling; RF, KNN, SVM, and RR were used for modeling and evaluation, and the prediction results of the optimal model were selected. The prediction results of the three data sources were put into the stacking integrated learning algorithm for regression prediction, and the decision-level fusion of cotton leaf nitrogen inversion results was obtained.

##### 2.3.1.3 Multi-source hybrid-fusion method

**Figure 6:**
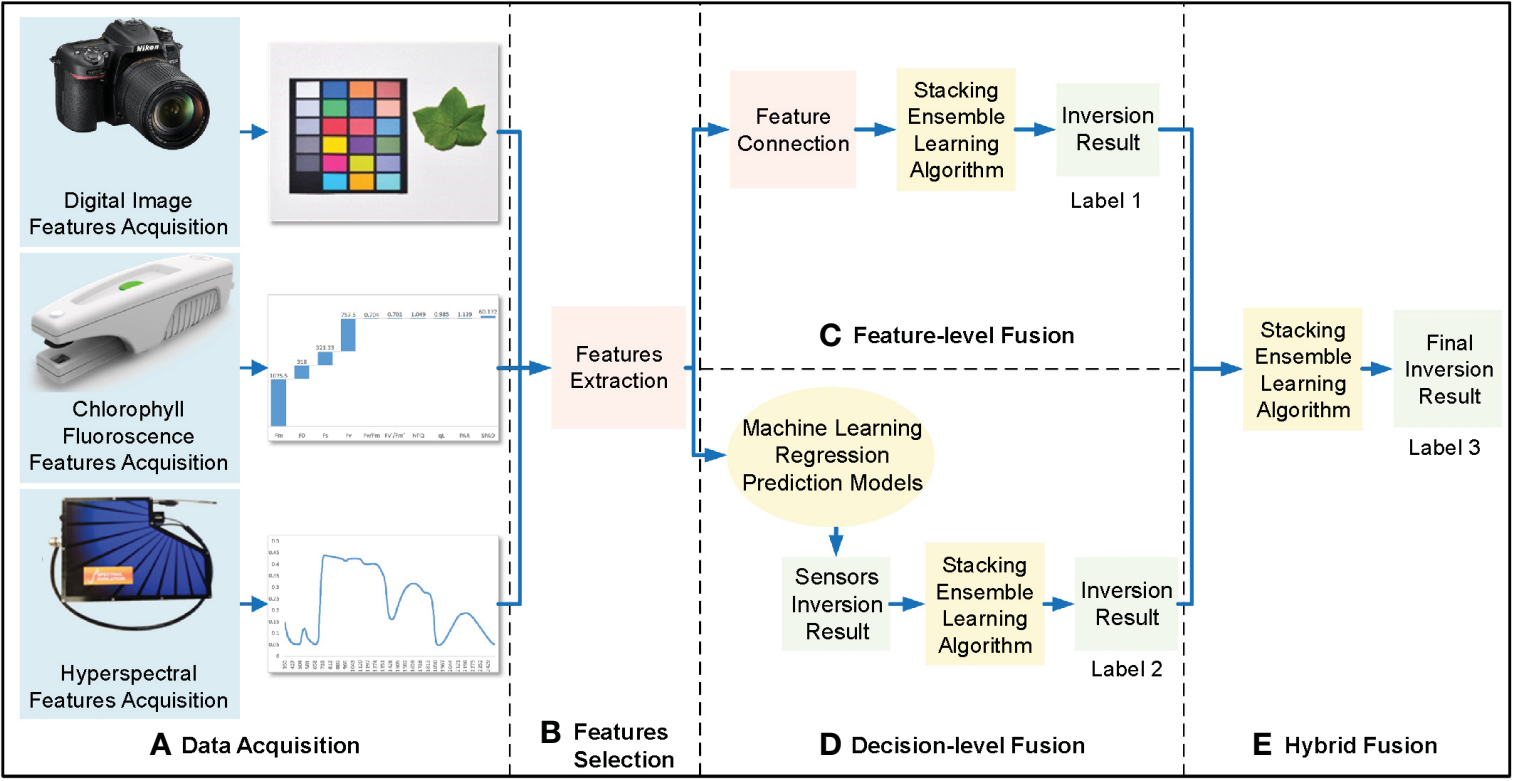
Hybrid-fusion framework of cotton leaf nitrogen inversion. **(A)** Data acquisition, **(B)** Features selection, **(C)** Regression modeling, **(D)** Feature-level fusion.

Hybrid fusion is similar to decision-level fusion; the feature-level and decision-level fusion results were fed second into the stacking integrated learning algorithm for regression prediction to obtain hybrid fusion cotton leaf nitrogen inversion results (**Figure 6**).

#### 2.3.2 Model evaluation

The determination coefficient (R^2^) and root-mean-square error (RMSE) were selected as evaluation indicators of the model. R^2^ indicates a deviation between the predicted and actual values; larger R^2^ values indicate better fitting models. RMSE analyzes the accuracy of the predicted and the actual values: smaller RMSEs indicate smaller deviations between the predicted and actual values and higher model accuracy. The calculation formulas for R^2^ and RMSE are as follows:


$$R^{2}=1-\frac{\sum_{i=1}^{n}{\left(x_{i}-{\bar{y}}_{i}\right)}^{2}}{\sum_{i=1}^{n}{\left(y_{i}-{\bar{y}}_{i}\right)}^{2}}$$

$$RMSE=\sqrt{\frac{\sum_{i=1}^{n}{\left(x_{i}-y_{i}\right)}^{2}}{n}}$$

where *x_i_* is the predicted value, *yi* is the actual value, $\bar{y_{i}}$ is the average of the actual values, and *n* is the number of samples available for validation.

## 3 Results and analysis

### 3.1 Screening of nitrogen-sensitive feature parameters based on different data sources

#### 3.1.1 Screening of hyperspectral feature parameters

The method in Section 2.2.2.1 was used to obtain the hyperspectral feature parameters. The results showed that there were approximately 10 infrared-sensitive feature bands of nitrogen content selected under the different treatments (**Table 1**), but with some differences due to the environment and variety. The sensitive feature band of processed spectral information is consistent between SG-SNV-Detrending and R processing, but the [lg(SG)]” processed spectral fluctuation amplitude is larger than that in R processing. This shows that different spectral preprocessing methods affect the selection of sensitive feature bands.

**Table 1:** Screening results of hyperspectral sensitive bands under different treatments.

Pretreatment	Feature Sensitive Bands
R	2212, 2225, 2224, 1253, 2222, 2307, 1704, 2235, 1724, 2213, 1728, 1702, 2337
[lg(SG)]”	1293, 1174, 1077, 1123, 1650, 1251, 1100, 1244, 1490, 1665
SG-SNV-Detrending	2178, 2213, 2212, 2214, 1725, 2197, 2196, 2195, 1728, 1726

#### 3.1.2 Screening of chlorophyll fluorescence feature parameters

**Figure 7:**
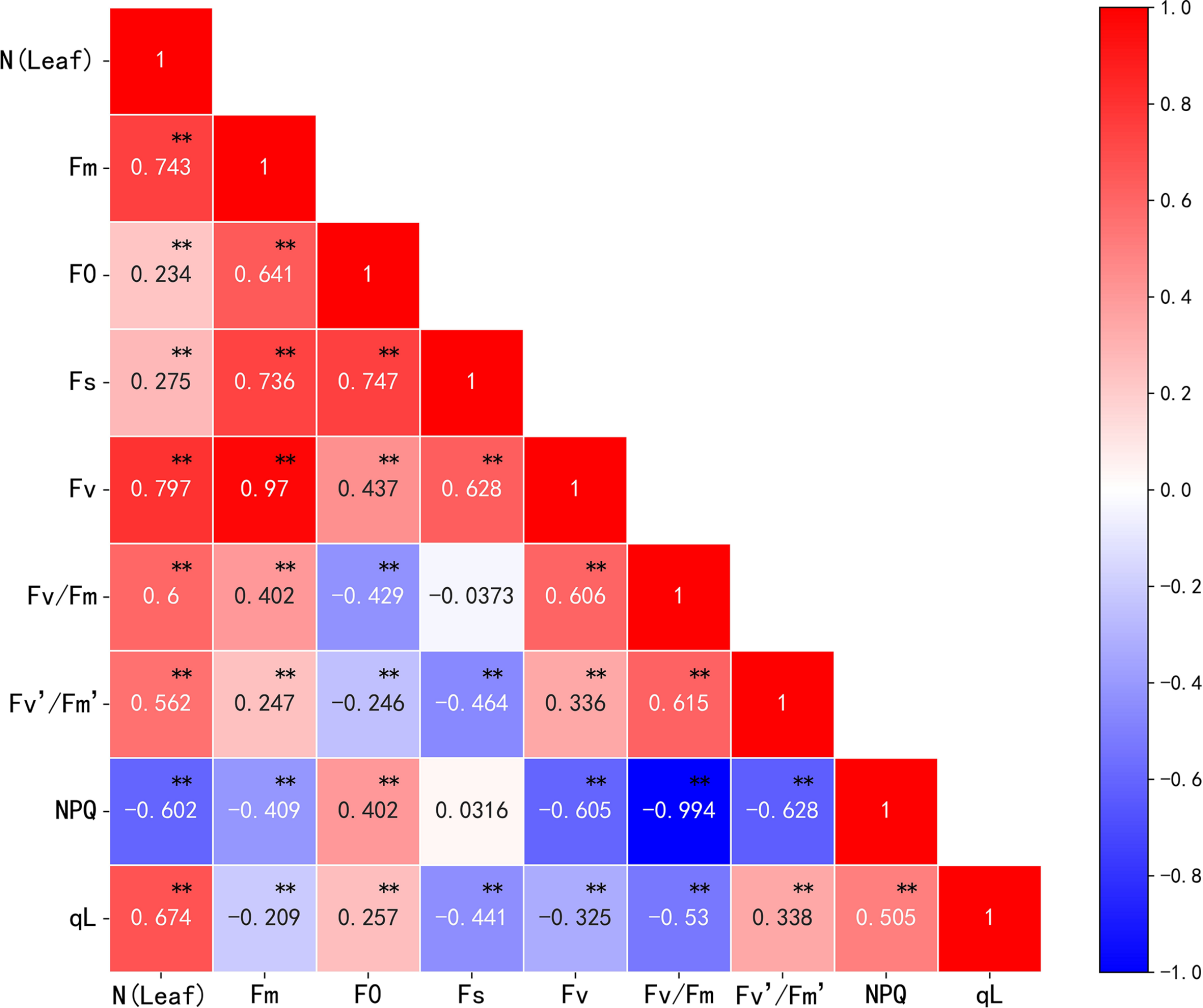
Correlation analysis of nitrogen content and chlorophyll fluorescence parameters in cotton leaves. *p< 0.05, **p< 0.01.

Nitrogen can alter the photosynthetic capacity of crop leaves; fluorescence, as a direct probe for photosynthesis, can be an indicator of crop nitrogen content. In this study, the correlation between chlorophyll fluorescence parameters and cotton leaf nitrogen content was analyzed. The results showed a significant correlation between the chlorophyll fluorescence parameters and cotton leaf nitrogen content (**Figure 7**). Fm, Fv, Fv/Fm, Fv’/Fm’, qL, and NPQ correlated with nitrogen content, and the absolute value of the correlation coefficient was greater than 0.5. All fluorescence parameters except NPQ correlated positively with cotton leaf nitrogen content. The correlation coefficients between Fm and Fs, Fm and Fv, F0 and Fs, and NPQ and Fv/Fm were 0.736, 0.970, 0.747, and -0.994, respectively. This shows a strong correlation between the corresponding parameters, high information overlap, and some multicollinearity. Therefore, principal component analysis (PCA) was used for linear transformation to remove feature redundancy and noise reduction. The principal component information loads (variance percentages) were 45.507%, 35.048%, 17.780%, 1.181%, 0.438%, 0.036%, 0.011%, and 2.819 × 10^-15^%. This indicates that the first, second, third, and fourth principal components carried more feature information, and the cumulative variance rate reached 99.516%. If the number of principal components continues to increase, the information load will fall below 1%, and the cumulative variance rate will no longer increase significantly. Therefore, the first four principal components were used as chlorophyll fluorescence features for subsequent modeling in this study.

#### 3.1.3 Screening of image feature parameters

**Figure 8:**
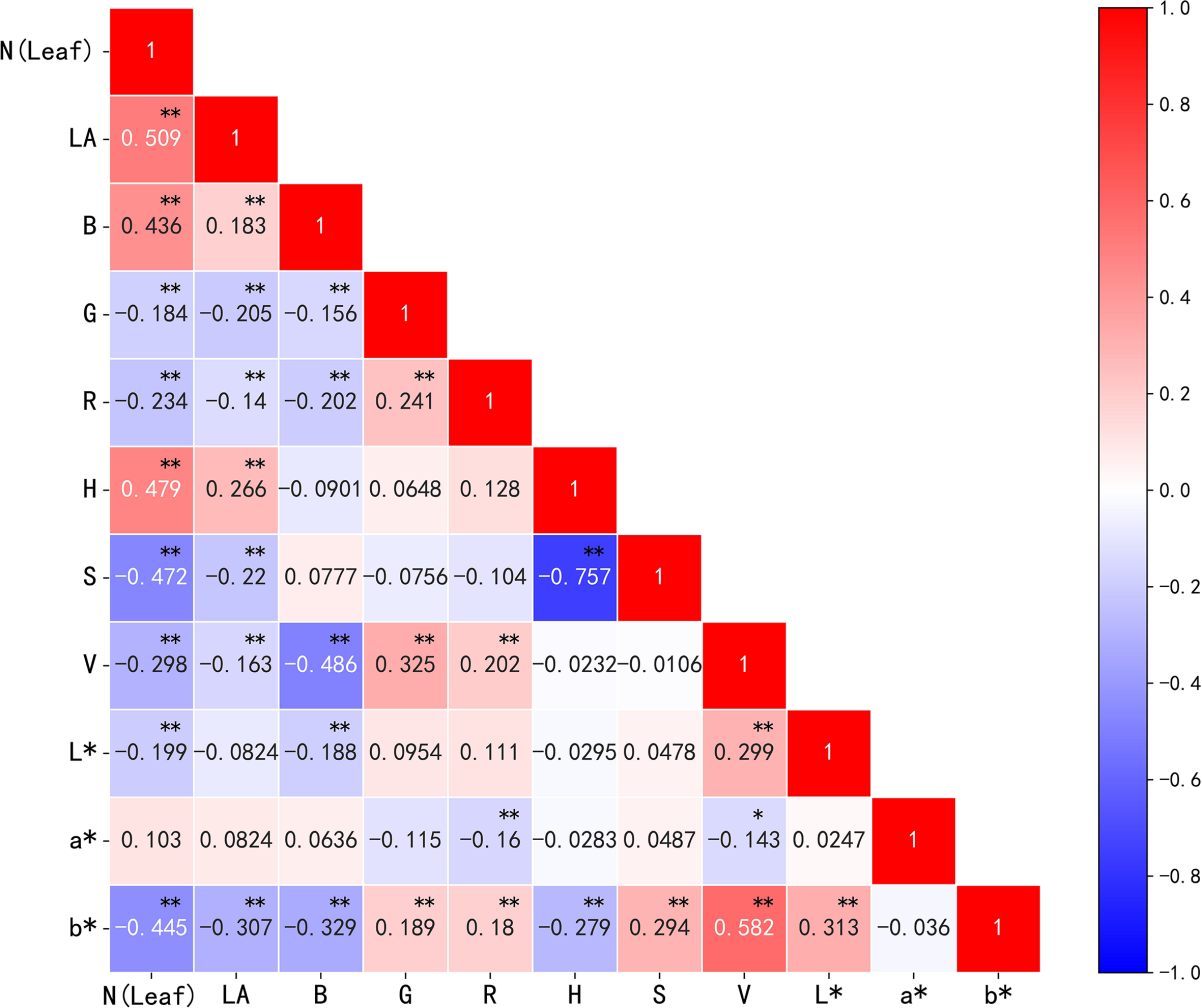
Correlation analysis of nitrogen content and image parameters in cotton leaves. *p< 0.05, **p< 0.01.

The correlations between image parameters (the first-order color moment in the three color spaces and the morphological parameter leaf area (LA)) and the nitrogen content in cotton leaves were analyzed. The results (**Figure 8**) showed that all parameters except the color parameter a* were significantly correlated. LA, B, H, and a* correlated positively with nitrogen content, whereas G, R, S, V, L*, and b* correlated negatively with nitrogen content. Where LA, B, H, S, and b* had a highly correlated relationship with nitrogen content, R^2^ reached 0.509, 0.436, 0.479, -0.472, and -0.445, respectively, and the correlation between the above parameters was low, indicating that the feature redundancy was low and the collinearity was weak; this means these parameters can be used as the image features for the subsequent modeling processing.

### 3.2 Cotton leaf nitrogen content estimation model based on single data source in fusion framework

#### 3.2.1 Cotton leaf nitrogen content estimation model based on hyperspectral features

**Figure 9:**
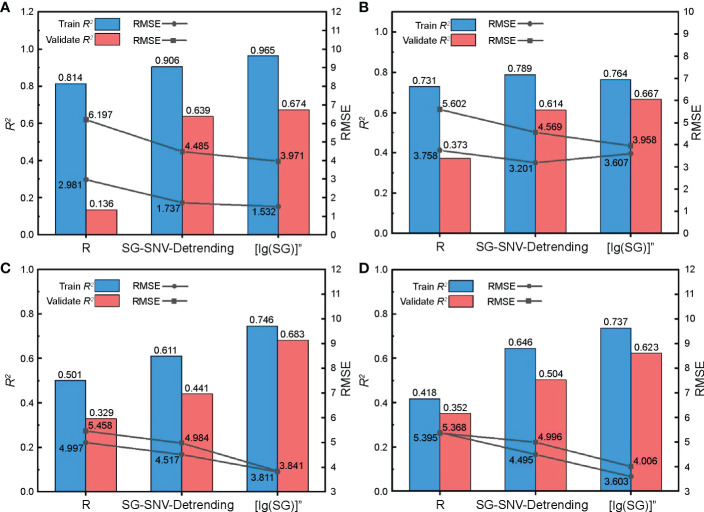
Validation of the cotton leaf nitrogen content estimation model based on hyperspectral features. Validation results of **(A)** random forest (RF), **(B)** K-nearest neighbor (KNN), **(C)** support vector machine (SVM), and **(D)** ridge regression (RR) models.

The spectral features obtained from Section 3.1.1 were substituted into supervised learning regression algorithms of different principles to model and evaluate the nitrogen content in cotton leaves. We used RF, KNN, SVM, and RR models to avoid duplication of effort due to weak differences between models. RF is a set of multiple decision trees, KNN performs regression using finding the nearest neighbors of the samples, SVM performs regression using the kernel method, and RR is an improved partial least squares regression. From the results of the model evaluation indices (**Figure 9**), the estimation accuracy of the sensitive feature bands based on R under four model training events was low, with an R^2^ value of 0.352, which is lower than that obtained from SG-SNV-Detrending and [lg(SG)]” preprocessing. Sensitive feature bands screened using [lg(SG)]” and SG-SNV-Detrending pretreatment were substituted into the four models, and R^2^ was approximately 0.4–0.7, which can be used for quantitative evaluation of nitrogen content. When constructing the RF model with [lg(SG)]” pretreatment, the training set R^2^ was 0.965, RMSE was 1.532, and the fit of the model was excellent. However, the validation set R^2^ and RMSE were lower than those of the SVM model under the same pretreatment, indicating that its generalization performance is inferior to that of the SVM model. By comprehensive comparison, the SVM model built with spectral features under the [lg(SG)]” pretreatment had an optimal estimation of cotton leaf nitrogen content, with a training set R^2^ and RMSE of 0.746 and 3.811, and a validation set R^2^ and RMSE of 0.683 and 3.841, respectively.

#### 3.2.2 Cotton leaf nitrogen content estimation model based on chlorophyll fluorescence features

**Figure 10:**
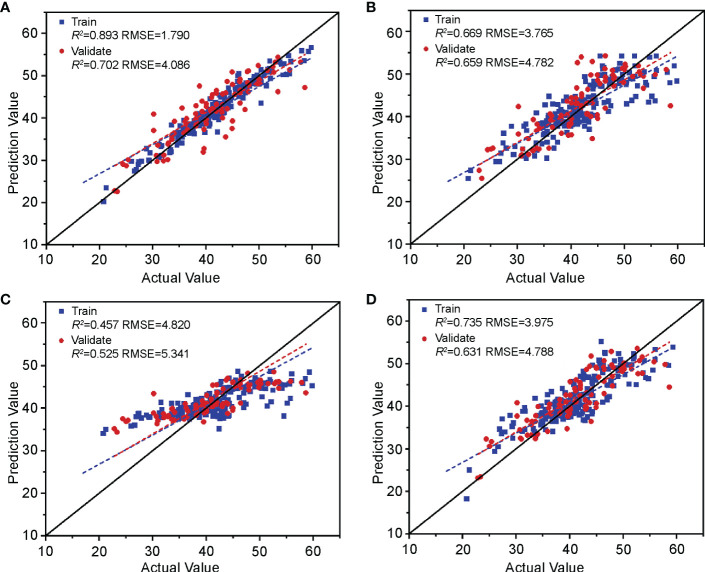
Validation of the cotton leaf nitrogen content estimation model based on chlorophyll fluorescence features. Validation results of **(A)** RF, **(B)** KNN, **(C)** SVM, and **(D)** RR models.

The model construction method is consistent with the hyperspectral estimation model in Section 3.2.1; that is, RF, KNN, SVM, and RR machine learning models were used to establish a relationship between the chlorophyll fluorescence features screened in Section 3.1.2 and the nitrogen content of cotton leaves. The results (**Figure 10**) show that the R^2^ of the RF, KNN, and RR training and validation sets were greater than 0.6, and the RMSE was less than 5, indicating that the above three models can estimate nitrogen content. The SVM had the effect of under-fitting. The training set R^2^ was 0.457, and the validation set R^2^ was 0.525. The model-learning ability was insufficient, and the generalization ability was weak. By comprehensive comparison, the R^2^ and RMSE of the estimation model of cotton leaf nitrogen content based on RF were optimal, with a training set R^2^ and RMSE of 0.893 and 1.790, and a validation set R^2^ and RMSE of 0.702 and 4.086, respectively, which can be used for cotton leaf nitrogen content estimation based on chlorophyll fluorescence features.

#### 3.2.3 Cotton leaf nitrogen content estimation model based on image features

**Figure 11:**
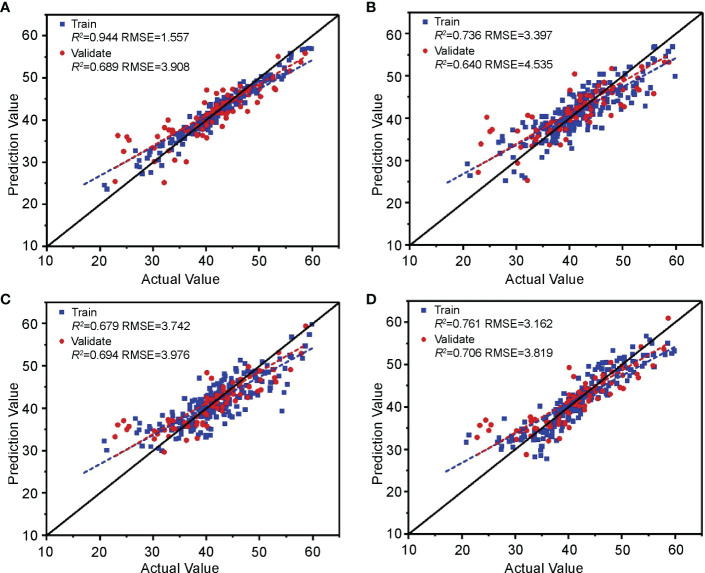
Validation of the cotton leaf nitrogen content estimation model based on image features. Validation results of **(A)** RF, **(B)** KNN, **(C)** SVM, and **(D)** RR models.

The above four machine learning models were established based on the image features selected in Section 3.1.3 and the cotton leaf nitrogen content. According to the evaluation results of each model training and validation set (**Figure 11**), the R^2^ of RF, SVM, and RR was approximately 0.7. The RMSE was approximately 3.9, indicating that the RF, SVM, and RR algorithms have excellent accuracy and stability in modeling and can reliably estimate the nitrogen content in cotton leaves, although the R^2^ of KNN was 0.64, and the RMSE was 4.535. By comprehensive comparison, the estimation model of cotton leaf nitrogen content based on RR was optimal, with a training set R^2^ and RMSE of 0.761 and 3.162, and a validation set R^2^ and RMSE of 0.706 and 3.819, respectively, which can be used for cotton leaf nitrogen content estimation based on image features.

### 3.3 Cotton leaf nitrogen content estimation model based on “image-spectrum-fluorescence” data fusions

#### 3.3.1 Cotton leaf nitrogen content estimation model based on feature-level fusion

**Figure 12:**
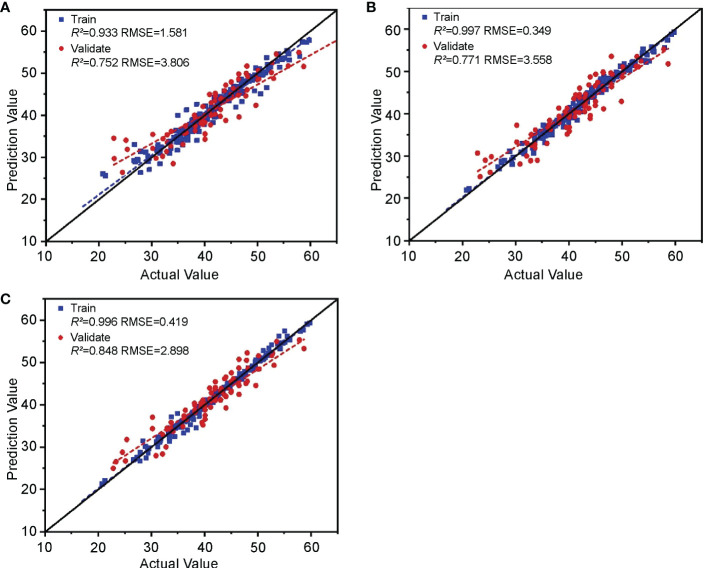
Validation of the cotton leaf nitrogen content estimation model based on “image-spectrum-fluorescence” data fusion. Validation results of **(A)** feature-level fusion, **(B)** decision-level fusion, and **(C)** hybrid-fusion models.

The feature parameters of hyperspectral, chlorophyll fluorescence, and digital images screened in Section 3.1 were cascaded. In feature-level fusion, after permutation, combination, and screening, the learners in stacking integrated learning obtained the optimal combination using RF and RR as base-learners and SVM as a meta-learner. It can be seen from the evaluation results (**Figure 12A**) that the training set R^2^ of the feature-level fusion model is 0.933 and the RMSE is 1.581, which shows that the goodness of fit of the model to the training set data is excellent, and that it has comprehensively learned the properties of the training set data. The validation set R^2^ was 0.752, and the RMSE was 3.806, indicating that the strong learning ability did not weaken its generalization, and the accuracy remained stable.

#### 3.3.2 Cotton leaf nitrogen content estimation model based on decision-level fusion

The prediction results of the three optimal models constructed using the hyperspectral, chlorophyll fluorescence, and digital image features in Section 3.2 were substituted into the decision-level fusion method for training and validation. Among them, the optimal combination of stacking integrated learning in decision-level fusion included KNN and RR as base-learners and RF as a meta-learner. (**Figure 12B**) The decision-level fusion model had a training set R^2^ and RMSE of 0.997 and 0.349, and a validation set R^2^ and RMSE of 0.771 and 3.558, respectively. Although the R^2^ value of the decision-level fusion training set is close to 1, the validation set R^2^ shows excellent model performance with no significant decline. This can be used for cotton leaf nitrogen content estimation. Combined with the RMSE, the model’s accuracy is slightly better than the feature-level fusion model.

#### 3.3.3 Cotton leaf nitrogen content estimation model based on hybrid fusion

The feature-level and decision-level fusion prediction results were substituted into the hybrid fusion for modeling, and training validation was conducted. Among them, the optimal combination of stacking integrated learning in hybrid fusion included KNN and RR as base-learners and RF as meta-learner. The results (**Figure 12C**) show that, in the hybrid-fusion model, the training set R^2^ and RMSE were 0.996 and 0.419, and the validation set R^2^ and RMSE were 0.848 and 2.898, respectively. Compared with the feature-level and decision-level fusion, the linear fitting relationship between the model’s actual and the predicted values was closer to a 1:1 line. Therefore, the hybrid-fusion model was the best among the cotton leaf nitrogen content estimation models based on “spectrum-fluorescence-image” data fusion.

### 3.4 Models performance comparison test

**Figure 13:**
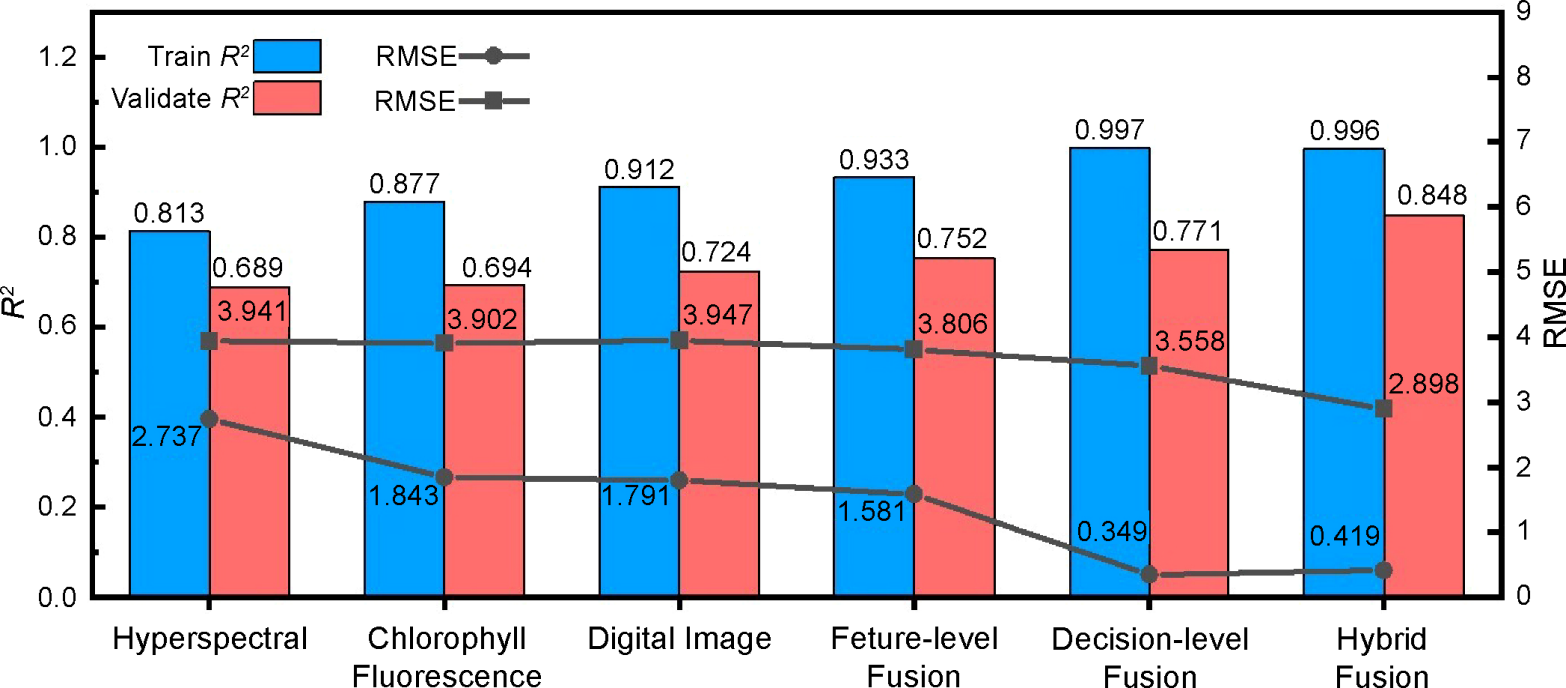
Comparison of the precision of the optimal model between multilevel data fusion and three single data sources.

The algorithm’s advantages enabled us to compare single data source models with multilevel fusion models in the same algorithm dimension by avoiding false improvement in fusion model accuracy. Therefore, the features of the spectrum, chlorophyll fluorescence, and image were substituted into the stacking integrated learning algorithm, and the best combination of learners was selected for training and validation, aiming to unify the algorithm that outputs the final prediction results in the fusion models. The comparison results are shown in **Figure 13**. Compared with the three single data source models, the feature-level fusion model validation set R^2^ improved by 2.8–6.3%, and the RMSE decreased by 4.7–8.2%. The decision-level fusion model validation set R^2^ increased by 2.5–3.6% and the RMSE decreased by 8.8–9.9%. The R^2^ of the hybrid-fusion model validation set increased by 12.4–15.9%, and the RMSE decreased by 25.7–26.6%. These results indicate that all multilevel data-fusion methods improved the accuracy of regression fitting of the three single data sources to different degrees. Among them, the accuracy of the feature-level and decision-level models in predicting the outcome, although improved compared with the single data source models, is not significant, as it can combine the prediction results of feature-level fusion and decision-level fusion with the real values for a second fit, greatly improving the evaluation indicators and achieving optimal accuracy and stability of the model. Therefore, the hybrid-fusion model can be applied for cotton leaf nitrogen content monitoring.

## 4 Discussion

Applying hyperspectral techniques to invert parameters, such as leaf nitrogen content, is one of the main tools for studying the nitrogen nutrient status of cotton. First, preprocessing and data transformation of the original feature spectra are important steps to effectively improve the accuracy of the model. Previous studies have shown that preprocessing of spectra can effectively eliminate noise and baseline drift and highlight the location of spectral feature bands (Arneberg et al., 2007). The accuracy of the models built using relevant studies (Nguyen and Lee, 2006; Li et al., 2016; Wang et al., 2020; Li et al., 2020b) using different forms of spectral transformations is higher than the models built using the original spectra. Feature screening mainly solves the collinearity problem of the spectrum, and wavelengths with less redundant information are selected for direct model construction, which is a simple, fast, and intuitive method easy to implement (Bai et al., 2018; Jia et al., 2019). In this study, the [lg(SG)]”-SVM model was the most accurate (training set R^2^ = 0.746, RMSE = 3.811, validation set R^2^ = 0.683, RMSE = 3.841). The original spectra can effectively extract the feature parameters and improve model accuracy using both transformation forms. To prevent overlap between the hyperspectral feature bands obtained from the experiment and the visible region acquired from the digital images, we used the first 10–13 near-infrared region-sensitive bands for modeling to ensure differences between the data sources, reduce the collinearity of each variable of the fusion model, and improve the accuracy and stability, and the selected sensitive bands were similar to those from previous studies (Niu et al., 2000; Xue et al., 2003).

Spectroscopic technology can be used to effectively monitor the nitrogen content of crops. However, spectral reflectance mainly reflects the concentration of plant biochemical components, is not sensitive to crop photosynthetic activity, and cannot directly reveal the photosynthetic physiological state of vegetation (McMurtrey et al., 2003; Ashourloo et al., 2014). Although chlorophyll fluorescence can provide information directly related to plant physiological functions (Smith et al., 2018), it has been widely used for crop growth, development, and nutrition monitoring in recent years. In this study, by analyzing the relationship between chlorophyll fluorescence parameters and nitrogen content in cotton leaves, we found that the correlations between Fm, Fv, Fv/Fm, Fv’/Fm’, qL, NPQ, and nitrogen content were stronger than the correlations between fluorescence parameters and nitrogen content of wheat plants established by Feng et al. (2012) and Zivcak et al. (2015). This indicated that different degrees of correlation exist between the fluorescence parameters and nitrogen content for different crops. Because of the strong multicollinearity among individual parameters, PCA was used to reduce the dimensionality, and variables with high information overlap were regrouped to reduce feature redundancy while retaining most of the valid information. The first four principal components were selected to establish a model for estimating the nitrogen content of cotton leaves based on chlorophyll fluorescence characteristics, and the results showed the best RF (training set R^2^ = 0.893, RMSE = 1.790, validation set R^2^ = 0.702, RMSE = 4.086); RF is an integration of multiple decision trees with high error balance and noise immunity and is widely used by researchers for crop phenotypic indicator monitoring with good modeling results (Han et al., 2019; Lu et al., 2019; Wang et al., 2021).

Digital image technology can identify the subtle differences between the surface layers of samples within the range of visible light. When collecting digital images of cotton leaves, it has been found that the influence of uncertain factors, such as the external environment and light intensity, is likely to lead to inaccurate and inconsistent image colors (You et al., 2020). In this study, the effect of these random errors on image color features was minimized by taking pictures in sufficient sunlight and correcting the standard color card. Huang et al. (2020) and Agarwal and Gupta (2018) proved that the fitting and generalization performance of a model built using combining multiple color spaces was better than that using a single color space. Therefore, in this study, the first moments of RGB, HSV, and L*a*b* color spaces and LA parameters were used to establish correlations with cotton leaf nitrogen content, extract as many of the relevant features of the image as possible from multiple angles, integrate more comprehensive information, and improve the accuracy of the inversion results of the subsequent fusion algorithm. All components of the RGB color space contain brightness information; therefore, they are vulnerable to external conditions; however, the hue and saturation in the HSV color space are separated from the value, and the color channels a* and b* in the L*a*b* color space are separated from the luminance, correcting the leaf color deviation in RGB color spaces to some extent. The color-calibration card is secondarily used to standardize the color of all leaves, avoiding large differences in the models’ parameters and results due to different external conditions, light, and other uncertain factors. Therefore, the model established in this study is more general. When inverting models of cotton leaf nitrogen content based on digital image features, the evaluation index value of RR was the best among the four machine learning models (training set R^2^ = 0.761, RMSE = 3.162, validation set R^2^ = 0.706, RMSE = 3.819). This shows that although the correlation between the filtered image features is low, there is also a weak linear relationship. The RR loss is unbiased in exchange for numerical stability; therefore, the fitting is more effective. In addition, RF and SVM showed good capability for estimating the nitrogen content based on image features.

Although data-fusion technology has developed rapidly, its application is mainly limited to urban planning, target classification, and recognition (Xing and Meng, 2018; Hoffmann et al., 2019; Zhao et al., 2021). Few studies on the inversion of agricultural parameters have achieved fusion only at the data and feature levels (Zhou et al., 2017; Li, 2018; Zhang, 2020; Jing et al., 2022), and the improvement of model accuracy and stability has been relatively limited. No multimodal and multilevel fusion regression model has been proposed for crop nutrition monitoring. In this study, a multilevel data-fusion model combining multiple machine learning and stacking integrated learning was built to accurately monitor the nitrogen content of cotton crops to obtain as many phenotypic structural, physiological, and biochemical features related to cotton nitrogen as possible through hyperspectral, chlorophyll fluorescence, and digital image data sources.

Out of the three data source features for modeling, feature-level fusion can obtain the highest level of complementary information (training set R^2^ = 0.933, RMSE = 1.581, validation set R^2^ = 0.752, RMSE = 3.806). Decision-level fusion can further train and learn from the results of individual decisions made using each data source (training set R^2^ = 0.997, RMSE = 0.349, validation set R^2^ = 0.771, RMSE = 3.558). Machine learning is adopted for independent modeling of each data source instead of stacking because: (1) the single data source features substituted into the stacking model do not significantly improve the accuracy; (2) the multilevel fusion model must consider running time and efficiency, and the stacking algorithm is used in this study to output the final result of each fusion framework; and (3) combining the three-node parallel-running stacking models with multiple nested stacking models increases the time consumption and the running load of the entire fusion model greatly. Therefore, the machine learning model is used for single data source modeling.

The hybrid-fusion method can integrate the training results of feature-level and decision-level fusion models, has significant anti-noise ability, and significantly improves the regression accuracy of nitrogen content inversion (training set R^2^ = 0.996, RMSE = 0.419, validation set R^2^ = 0.848, RMSE = 2.898). The precision of the fused models is better than the single data source models under the same algorithm, and the precision of the hybrid-fusion model is optimal. This shows the superiority of data fusion using spectral data, chlorophyll fluorescence parameters, and digital images to estimate cotton nitrogen content. However, the consequent limitation is the increase in data acquisition costs. Different data acquisition devices can be integrated to solve this problem and achieve portable, synchronous, and efficient data acquisition. Follow-up research can apply the above methods to monitoring nutrient elements at different scales during different growth periods of crops, providing a reference and ideas for implementing precision agriculture. With changes in the growth period and other factors, the nitrogen nutrition status of cotton leaves will also change, leading to differences in the parameters related to nutritional status extracted from each data source. Therefore, it is necessary to adjust the method of optimizing feature screening and model construction while continuously enriching the experimental data.

## 5 Conclusion

In this study, three data sources—hyperspectral, chlorophyll fluorescence, and digital camera—were used to monitor cotton leaf nitrogen content, screen the features of each data source, and establish multilevel data-fusion models. The following conclusions were drawn:

(1) The original spectral information was pre-treated using different spectral transformation methods. Bands that correlated significantly with the nitrogen content of cotton leaves were selected; each round of processing yielded approximately 10 near-infrared bands as spectral-based features. Correlation analysis between chlorophyll fluorescence parameters and nitrogen content revealed significant collinearity between some parameters, and the first four principal components based on fluorescence were selected as features using PCA. Meanwhile, the correlation between the extracted image parameters and the nitrogen content was analyzed, and the parameters with strong correlation, low redundancy and low collinearity were selected as the features based on digital images.

(2) The evaluation index values were optimal when the spectral data were processed using [lg(SG)]”-SVM to estimate nitrogen content, the chlorophyll fluorescence features substituted into RF were used to build the nitrogen content estimation model, and the digital image features substituted into RR were used to build the nitrogen content estimation model. The three optimal estimation models of data sources were used for the steps in the decision-level fusion framework.

(3) This study combined the information obtained from the three data sources to build multilevel data-fusion models. Compared with the single data source models under the same algorithm, multilevel data-fusion models had improved accuracy and stability. They can improve the accuracy and stability of cotton leaf nitrogen inversion to varying degrees, and the hybrid-fusion model had the best results. This provides a feasible method and approach precisely monitoring crop nitrogen nutrition.

## Data availability statement

The original contributions presented in the study are included in the article/supplementary material. Further inquiries can be directed to the corresponding authors.

## Author contributions

Conceptualization, SQ, YD, and ZZho. Methodology, SQ. Validation, SQ and QY. Formal analysis, SQ, HW, FX and ZZho. Investigation, SQ and YD. Resources, SQ, YD and MZ. Data curation, SQ and MZ. Writing—original draft preparation, SQ. Writing—review and editing, SQ, LZ and ZZha. Funding acquisition, ZZha, LZ and XL. All authors contributed to the article and approved the submitted version.
